# Early sleep after action observation plus motor imagery improves gait and balance abilities in older adults

**DOI:** 10.1038/s41598-024-53664-2

**Published:** 2024-02-07

**Authors:** Federico Temporiti, Elena Galbiati, Francesco Bianchi, Anna Maria Bianchi, Manuela Galli, Roberto Gatti

**Affiliations:** 1 Physiotherapy Unit, Humanitas Clinical and Research Center - IRCCS, via Manzoni 56, Rozzano, Milan Italy; 2 Department of Electronic, Information and Bioengineering, Politecnico Di Milano, via Ponzio 34, Milano, Milan Italy; 3 Department of Biomedical Sciences, Humanitas University, via Rita Levi Montalcini 4, Pieve Emanuele, Milan Italy

**Keywords:** Neuroscience, Sensorimotor processing

## Abstract

Action observation plus motor imagery (AOMI) is a rehabilitative approach to improve gait and balance performance. However, limited benefits have been reported in older adults. Early sleep after motor practice represents a strategy to enhance the consolidation of trained skills. Here, we investigated the effects of AOMI followed by early sleep on gait and balance performance in older adults. Forty-five older adults (mean age: 70.4 ± 5.2 years) were randomized into three groups performing a 3-week training. Specifically, AOMI-sleep and AOMI-control groups underwent observation and motor imagery of gait and balance tasks between 8:00 and 10:00 p.m. or between 8:00 and 10:00 a.m. respectively, whereas Control group observed landscape video-clips. Participants were assessed for gait performance, static and dynamic balance and fear of falling before and after training and at 1-month follow-up. The results revealed that early sleep after AOMI training sessions improved gait and balance abilities in older adults compared to AOMI-control and Control groups. Furthermore, these benefits were retained at 1-month after the training end. These findings suggested that early sleep after AOMI may represent a safe and easy-applicable intervention to minimize the functional decay in older adults.

## Introduction

The deterioration of body structures and functions induced by ageing has been reported to impair postural stability, leading to gait and balance deficits responsible for functional independence loss and increased risk of falling^1^. The physiopathology of gait and balance impairments in older adults includes peripheral factors, such as decreased lower limb range of motion, muscle mass and sensory receptors loss and progressive peripheral vestibular deficits^2–5^. Furthermore, sensorimotor system alterations and high-order cognitive functions decline have been also reported as central nervous system-related mechanisms responsible for gait and balance impairments in older adults^6,7^. In this scenario, motor rehabilitation represents an effective intervention to enhance gait and balance and prevent the subsequent decay in terms of functional performance^8,9^. Balance exercises should be challenging and tailored on participants’ abilities, requiring a certain level of supervision in order to be effective and safe at the same time^9^. In fact, literature data have reported the superiority of a supervised home-based balance training on gait and balance abilities, when compared to an unsupervised exercises regimen in older adults^10,11^. Therefore, approaches aimed at boosting the effects of unsupervised balance training or addressed to improve gait and balance without exposing participants to risk of fall may assume relevance in older adults.

Action observation and motor imagery represent rehabilitative approaches to improve motor and functional abilities in the absence of action execution^12^. Action observation implies the observation of video-clips including motor contents in order to exploit the ability of the mirror neuron system to recruit motor areas responsible for the representation of observed actions, while motor imagery includes the mental simulation of observed tasks allowing for a wider resonance of the cortical sensorimotor network^12,13^. In addition, higher benefits have been reported in terms of motor learning when the aforementioned approaches are merged into an action observation plus motor imagery intervention (AOMI)^12^. When considering the neurophysiological rationale of the application of this approach to enhance gait and balance abilities, studies have reported that AOMI of dynamic balance tasks activates the dorsal and ventral premotor cortices, supplementary and primary motor areas, basal ganglia and cerebellum, revealing a substantial overlap with the brain network involved in motor execution^14,15^. Moreover, electroencephalographic investigations have demonstrated a modulation of brain activity in the alpha and beta bands of sensorimotor and anterior cingulate cortices during AOMI of a walking task^16^. Such phenomenon has been also reported to be dependent by walking phases, showing the AOMI ability to emulate the cortical activity induced by actual walking^16^. In addition, an increase in corticospinal excitability has been also reported during AOMI of walking, although this effect seems to be transient after a single session^17,18^. Interestingly, the aforementioned modulations in terms of brain activation pattern and corticospinal excitability have been reported to be higher during AOMI, when compared to action observation and motor imagery alone^14–17^.

The effects of AOMI training on gait and balance performance have been poorly investigated in older adults. In addition, limited AOMI benefits have been found in older adults, although studies have demonstrated that the physiological mirror neuron system activity does not reveal substantial age-related changes^19,20^. A single study reported that a 4-week AOMI training decreased postural sway during a perturbation balance task in older adults. However, no significant differences were found in favor of AOMI, when compared to a control group who performed no intervention^21^.

In this scenario, strategies aimed at further enhancing motor learning induced by non-physical approaches such as AOMI may play a key role in older adults, especially when improvements in gait and balance abilities without exposing subjects to risk of fall represent the goal of the rehabilitative intervention. Early sleep after motor practice has been reported as a viable strategy to improve the performance of trained motor skills, thanks to its ability to promote an offline consolidation of newly acquired memory traces^22^. Sleep-dependent consolidation processes of motor learning has been also demonstrated in older adults, as long as the proposed training induces a sufficient strength in the encoding of motor memory traces^23^. In these subjects, cortico-striatal neural activity patterns in specific phases of non-rapid eye movement sleep have been described during electrophysiological investigations and related to consolidation of motor memory traces^24^.

To date, sleep-dependent effects have been mainly investigated after a training based on motor practice^23,25^. Moreover, literature data have investigated upper limb dexterity changes induced by the observation with or without imagination of motor tasks, revealing higher benefits when the training sessions were followed by an early sleep-window^26–29^. Therefore, when considering the current background, it is reasonable to speculate that sleep might enhance the acquisition of trained motor skills such as gait and balance abilities, when occurring immediately after AOMI training sessions in older adults. Thus, the current study aimed at investigating the effects of early sleep after AOMI on gait and balance performance in older adults.

## Methods

### Participants

Forty-five older adults were enrolled between May and December 2022 according to the following inclusion criteria: age between 65 and 85 years, ability to walk independently without aids and right lower limb dominance according to Van Melick criteria (see Statistical analysis subsection for sample size estimation procedures)^30^. Exclusion criteria were diagnosis of neurological or musculoskeletal conditions able to affect gait and balance abilities, documented sleep disorders such as insomnia, obstructive sleep apnea syndrome, REM or non-REM behavior disorders, use of medications able to alter the physiological sleep pattern, psychiatric disorders and Mini Mental State Examination score lower than 25 points. Moreover, subjects performing job or recreational activities requiring nocturnal sleep deprivation or advanced gait and balance skills were also excluded. Participants’ characteristics are shown in the Table 1. The study was carried out at the Motion Analysis Lab of Humanitas Clinical and Research Center, Milan, Italy. All methods agree with the relevant guidelines and regulations, a written informed consent was obtained from all participants or their legal guardians, and the study protocol was approved by the Humanitas Clinical and Research Center Ethical Committee (n. CLF22/01, February 2022) and registered on ClinicalTrials.gov (NCT05393700, 26/05/2022). Informed consent from participants or their legal guardians for publication of identifying information/images in an online open-access publication was also obtained.

**Table 1:** Characteristics of study participants. Data are shown as mean and standard deviation, while the number of sleep hours per night and Pittsburgh Sleep Quality Index (PSQI) are also reported as median and range split for participants’ gender.

	AOMI-sleep	AOMI-control	Control	*p*-value (F or X^2^ values)
Age [years]	70.4 ± 4.8	68.5 ± 3.8	72.3 ± 6.4	0.129 (2.155)
Gender [M/F]	12/3	7/8	6/9	0.061 (5.580)
BMI [kg/m^2^]	25.7 ± 3.5	27.7 ± 4.0	25.7 ± 4.5	0.454 (0.804)
Lower limb length [cm]	87.3 ± 5.5	86.9 ± 3.6	86.0 ± 4.2	0.331 (1.134)
MMSE [points]	28.0 ± 1.1	27.3 ± 1.1	27.6 ± 1.2	0.270 (1.350)
KVIQ visual score [points]	38.3 ± 12.4	39.3 ± 8.2	33.0 ± 13.8	0.292 (1.270)
KVIQ kinaesthetic score [points]	18.9 ± 11.9	21.1 ± 9.7	18.9 ± 12.4	0.840 (0.175)
EQ5D utility score [%]	86.0 ± 9.9	76.9 ± 14.7	86.9 ± 10.8	0.052 (3.174)
EQ5D-VAS [points]	77.7 ± 9.0	74.7 ± 16.8	74.5 ± 16.2	0.795 (0.231)
MES [points]	62.7 ± 6.7	59.1 ± 7.3	60.3 ± 6.1	0.349 (1.080)
Sleep hours [hours/night]	7.0 ± 0.8 M: 7.1 (3.4), F: 6.7 (0.1)	7.3 ± 1.0 M: 8.2 (2.9), F: 7.1 (3.5)	7.2 ± 0.9 M: 6.6 (3.0), F: 7.0 (2.9)	0.707 (0.349)
PSQI [points]	4.5 ± 2.5 M: 4.0 (7.0), F: 6.0 (3.0)	6.0 ± 4.2 M: 5.0 (9.0), F: 6.0 (12.0)	4.9 ± 2.2 M: 3.5 (5.0), F: 5.0 (7.0)	0.383 (0.981)

*AOMI* Action observation plus motor imagery, *M* male, *F* female, *MMSE* Mini-Mental Sate Examination, *KVIQ* Kinesthetic Visual Imagery Questionnaire, *MES* Morningness-Eveningness Scale, *EQ5D* European Quality of Life 5-Dimension, *PSQI* Pittsburgh Sleep Quality Index.

### Study design and intervention

This was a three-armed single-blind randomized controlled study. Participants’ eligibility was assessed by an independent researcher blinded to the randomization list in order to ensure the allocation concealment. After the enrollment, participants were randomized into AOMI-sleep (n = 15), AOMI-control (n = 15) or Control (n = 15) groups through a simple computer-generated random sequence. After randomization, all participants observed 12-min video-clips, 4 times per week for 3 weeks (12 sessions). AOMI-sleep and AOMI-control groups observed and imagined daily tasks performed in standing posture and requiring advanced gait and balance abilities. Each training session included 3 tasks and participants performed 3 min of action observation followed by 1 min of visual motor imagery for each task. Visual stimuli adopted for action observation consisted of video-clips including gait and balance tasks delivered in third-person perspective from a lateral or/and frontal points of view. The actors’ gender and age bracket were congruent with those of the observers and the complexity of motor contents progressively increased over the 3 weeks of training^31^. A graphic illustration of the stimuli are shown in Supplementary material 1. Specifically, the following written instructions were given to participants on the screen: “Observe the task carefully focusing on how the actions are executed without performing any movement during observation”. After observation, motor imagery sessions started with a black frame on the screen associated with the following written request: “Imagine seeing yourself while performing the task just observed without performing any movement during imagination”^32^. Motor imagery was applied in third-person perspective and visual modality, based on literature data suggesting that visual motor imagery is easier to perform than kinesthetic motor imagery, especially when motor contents consist of whole-body complex movements, such as gait and balance tasks included in the current AOMI training^33,34^. Moreover, the use of the easiest modality of motor imagery was also driven by the unsupervised regimen of the training. AOMI-sleep and AOMI-control groups performed the same intervention, and the only difference was that AOMI-sleep group performed the training sessions between 8:00 and 10:00 p.m., whereas AOMI-control group underwent AOMI between 8:00 and 10:00 a.m. Control group observed a landscape video-clips between 8:00 and 10:00 p.m. Participants were instructed on the training by a researcher not involved in the assessment procedures. Moreover, they were asked to avoid daytime sleep during the training period and the treatment adherence was ensured through a daily phone call. Finally, participants were instructed to fill in a diary sheet reporting the timing of training sessions execution and the number of sleep hours per night.

### Assessment

Participants were assessed by a researcher blinded to group allocation at baseline (T0), training end (T1) and 1 month after the training end (T2).

The primary outcome was the 10-Meter Walk Test (10MWT) performed at self-paced and maximum speed for gait performance assessment. During the test, participants walked along a 10-m walkway at comfortable speed and as quickly as possible. The initial and final 2 m of the walkway were adopted for acceleration and deceleration, and the performance was timed with a stopwatch in order to compute the gait speed expressed in m/s. Two trials were administered for self-paced and maximum speed conditions and the average score for each condition was used for data analysis.

The secondary outcomes included the Timed Up and Go test (TUG) for mobility, the Four Step Square Test (FSST) and the Lower Quarter Y-Balance Test (LQYBT) with the left (L) and right (R) lower limbs for dynamic balance, the analysis of center of pressure kinematics during standing with open and closed eyes for static balance, and Fall Efficacy Scale (FES-I) for the participants’ concern about falling. During TUG, participants rose from a chair, walked at self-paced speed for 3 m, turned and walked back to the chair in order to sit down again. After a familiarization trial, two trials were performed. The performance was timed with a stopwatch and the best trial was included in data analysis. The FSST consisted of four sticks placed on the ground at an angle of 90 degrees to each other in order to create four squares. Starting from the top left square, participants had to step in each square moving in clockwise and counterclockwise directions as quickly as possible. They were asked to maintain the body facing forward during the test and avoid touching the sticks during the movement. The performance was timed with a stopwatch and the best scores of two trials was used for data analysis. The LQYBT included a stance platform connected with three pipes oriented in anterior (ANT), posterior-medial (PM) and posterior-lateral (PL) directions and creating a Y-shape. Participants performed three single-leg squats in order to reach with the non-stance limb the farthest point along each direction. Prior to test performance, the middle point of the stance-foot was placed at the Y-shape center, while the contralateral foot was placed 10 cm aside. After three familiarization trials, three trials with the left and right limbs were performed and the average score for each direction was used for data analysis, expressed as a percentage of lower limb length. Static balance was assessed during two 30-s quiet standing tasks. During the first task, participants had to maintain the standing posture with open eyes looking at a fixed point placed at 2-m distance, while the second task consisted of the maintenance of standing posture with closed eyes. Participants stood barefoot with arms crossed over the chest and feet placed parallel with heels at 5 cm apart. Center of pressure kinematics was detected using a force platform (P-6000, BTS, Italy). Raw data were sampled at 200 Hz and down-sampled at 20 Hz. Subsequently, center of pressure range in anterior–posterior (Range-AP) and medial–lateral (Range-ML) directions and total path length (TOT-PL) were calculated and normalized to participants’ height, according to literature^35^. The analysis was performed using Smart Analyzer software, BTS, Italy. At the end of static balance assessment, subjects filled in the FES-I, which consisted of a 16-item questionnaire where each item is scored on a 4-point scale. The final score ranges from 16 to 64 points, and higher score indicates lower concern about falling.

At baseline, participants were also assessed for motor imagery capabilities through the Kinesthetic and Visual Imagery Questionnaire 10 items (KVIQ-10), the individual chronotype using the Morningness-Eveningness Questionnaire (MEQ), perceived quality of life with the European Quality of Life 5-Dimension (EQ5D descriptive system and EQ-5D-VAS) and cognitive status with the Mini Mental State Examination. Finally, participants’ sleep quality during the training period was assessed through the Pittsburg Sleep Quality Index (PSQI) at T1, and the number of sleep hours per night was also collected.

### Statistical analysis

Sample size was estimated a-priori based on the 10MWT as primary outcome. Considering a Minimal Detectable Change (MDC_95_) of 0.13 m/s between AOMI-sleep and AOMI-control groups at T1, standard deviation of 0.14 m/s, 80% of power and alpha error of 5%, 15 participants were required for each group^36^.

Data were checked for normality using the Kolmogorov–Smirnov test. Univariate Analysis of Variance or Chi-Square test were adopted to investigate between-group differences in terms of participants’ characteristics at baseline (KVIQ-10, MEQ and EQ5D), sleep quality (PSQI) and sleep hours per night (number) during the training period. A 3 × 3 General Linear Model with Time as within-subjects factor and Group as between-subjects factor was used to assess between-group differences over time in terms of outcome measures (10MWT, TUG, FSST, LQYBT, center of pressure parameters and FES-I). When significant interactions or main effects occurred, Bonferroni post-hoc tests were used to investigate between-group differences at each timepoint and within-group differences among the three time-points. The effect size between the three groups was also calculated and expressed as mean difference (MD) and 95% confidence interval (CI_95_).

## Results

All participants completed the baseline and post-treatment evaluation sessions. No dropouts occurred and none of the participants continued the training after T1 assessment. No between-group differences were found for participants’ characteristics and outcome measures at baseline. Moreover, the number of sleep hours per night during the training period and PSQI score revealed no between-group differences (Table 1).

A Time by Group interaction, Group and Time effects were found for 10MWT at self-paced and maximum speed, TUG, FSST and R-LQYBT in ANT direction. A Group effect was detected for L-LQYBT in ANT direction, while Time effects were found for L-LQYBT in ANT direction, and L-LQYBT and R-LQYBT in PL directions. A Time by Group interaction was found for Range-ML during standing with open eyes, whereas no Time by Group interactions, Group or Time effects were found for other center of pressure parameters and FES-I (Tables 2 and 3).

**Table 2:** Between-group differences over time for gait performance outcomes (3 × 3 General Linear Model with Bonferroni post-hoc analysis).

	AOMI-sleep			AOMI-control			Control			*p*-value Time Factor (F-value)	*p*-value Group Factor (F-value)	*p*-value Time x Group interaction (F-value)
	T0	T1	T2	T0	T1	T2	T0	T1	T2			
Gait performance												
10MWT self-selected speed [m/s]	1.35 ± 0.13	1.50 ± 0.18*	1.53 ± 0.18*	1.28 ± 0.14	1.39 ± 0.15*	1.39 ± 0.11*	1.31 ± 0.20	1.31 ± 0.18	1.27 ± 0.17	** < 0.001** **(21.439)**	**0.018** **(4.420)**	** < 0.001** **(9.236)**
10MWT maximum speed [m/s]	1.77 ± 0.20	2.15 ± 0.25*	2.14 ± 0.31*	1.63 ± 0.21	1.78 ± 0.17*	1.74 ± 0.16*	1.70 ± 0.24	1.65 ± 0.25	1.60 ± 0.24	** < 0.001** **(47.798)**	** < 0.001** **(10.846)**	** < 0.001** **(41.138)**
TUG [s]	8.3 ± 1.1	6.9 ± 1.2*	6.4 ± 0.9*	8.3 ± 1.1	7.9 ± 0.8	7.9 ± 0.8	9.1 ± 1.7	9.3 ± 2.2	9.5 ± 1.8	** < 0.001** **(11.604)**	** < 0.001** **(10.335)**	** < 0.001** **(11.483)**

Data are shown as mean and standard deviation.

*AOMI* Action observation plus motor imagery, *10MWT* 10-Meter Walk Test, *TUG* Timed Up and Go.

Symbols: * *p* < 0.05 compared to T0 of the same group.

Significant *p*-values are shown in bold text.

**Table 3:** Between-group differences over time for dynamic balance and concern about falling outcomes (3 × 3 General Linear Model with Bonferroni post-hoc analysis). Data are shown as mean and standard deviation.

	AOMI-sleep			AOMI-control			Control			*p*-value Time Factor (F-value)	*p*-value Group Factor (F-value)	*p*-value Time x Group interaction (F-value)
	T0	T1	T2	T0	T1	T2	T0	T1	T2			
Dynamic balance												
FSST [s]	10.1 ± 1.6	7.9 ± 1.2*	7.7 ± 1.3*	10.4 ± 2.4	9.7 ± 1.8	9.8 ± 1.6	10.2 ± 1.5	10.3 ± 2.0	10.7 ± 2.3	** < 0.001** **(16.101)**	**0.011** **(5.066)**	** < 0.001** **(13.643)**
Right LQYBT-ANT [%]	78.2 ± 8.8	84.7 ± 10.7*	87.4 ± 14.5*	73.4 ± 8.3	75.1 ± 7.6	72.8 ± 7.4	75.9 ± 10.6	73.9 ± 11.0	77.0 ± 15.1	**0.036** **(3.699)**	**0.021** **(4.234)**	**0.006** **(4.243)**
Right LQYBT-PL [%]	76.5 ± 13.7	90.2 ± 14.7*	91.4 ± 20.2*	75.0 ± 15.1	78.2 ± 15.8	78.2 ± 15.1	77.9 ± 19.3	80.4 ± 18.7	82.4 ± 20.1	** < 0.001** **(11.183)**	0.305 (1.223)	**0.027** **(3.069)**
Right LQYBT-PM [%]	81.7 ± 12.9	92.2 ± 14.4*	93.7 ± 18.5*	80.5 ± 13.7	82.4 ± 13.4	79.7 ± 12.3	87.9 ± 18.8	83.8 ± 18.5	84.7 ± 18.9	0.079 (2.610)	0.322 (1.165)	** < 0.001** **(7.483)**
Left LQYBT-ANT [%]	79.5 ± 9.9	85.2 ± 11.1*	86.1 ± 15.6*	72.9 ± 7.0	75.2 ± 9.0	73.4 ± 9.2	77.3 ± 7.5	78.2 ± 10.9	78.0 ± 15.1	**0.042** **(3.708)**	**0.035** **(3.619)**	0.208 (1.558)
Left LQYBT-PL [%]	77.4 ± 13.8	85.4 ± 15.0*	89.9 ± 19.2*	72.6 ± 17.9	75.9 ± 10.8	76.2 ± 16.3	79.8 ± 16.3	81.1 ± 16.5	85.6 ± 19.0	** < 0.001** **(11.031)**	0.220 (1.571)	0.145 (1.801)
Left LQYBT-PM [%]	84.6 ± 11.7	93.0 ± 13.2	91.8 ± 18.8	82.4 ± 14.3	82.2 ± 12.9	82.7 ± 15.0	87.6 ± 12.8	86.9 ± 15.3	88.0 ± 19.7	0.108 (2.397)	0.353 (1.068)	0.060 (2.506)
Concern about falling												
FES-I [points]	20.3 ± 2.6	20.5 ± 4.5	20.3 ± 5.5	22.1 ± 6.3	22.0 ± 5.2	21.3 ± 4.2	19.0 ± 4.9	18.6 ± 2.3	18.5 ± 2.7	0.642 (0.323)	0.101 (2.419)	0.940 (0.115)

*AOMI* Action observation plus motor imagery, *FSST* Four Square Step Test, *LQYBT* Lower Quarter Y-Balance Test, *ANT* anterior, *PL* posterior-lateral, *PM* posterior-medial, *FES* Fall Efficacy Scale.\

Symbols: *p < 0.05 compared to T0 of the same group.

Significant *p*-values are shown in bold text.

**Figure 1:**
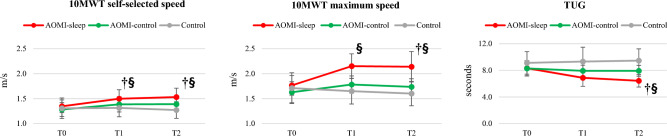
Between-group differences over time for gait performance outcomes (10-Meter Walk Test—10MWT and Timed Up and Go—TUG) are shown. Data are presented as mean (dots) and standard deviation (bars), and symbols († and §) represent significant differences between AOMI-sleep and AOMI-control groups and between AOMI-sleep and Control groups, respectively.

**Figure 2:**
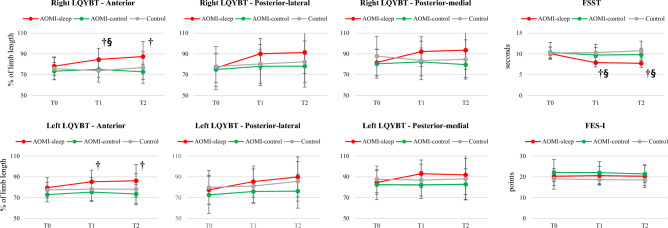
Between-group differences over time for dynamic balance outcomes (Right and Left Lower Quarter Y-Balance Test—LQYBT and Four-Square Step Test—FSST) and concern about falling (Fall Efficacy Scale—FES-I) are shown. Data are presented as mean (dots) and standard deviation (bars), and symbols († and §) represent significant differences between AOMI-sleep and AOMI-control groups and between AOMI-sleep and Control groups, respectively.

Between-group post-hoc analysis revealed better 10MWT at maximum speed, FSST and R-LQYBT in ANT direction in favor of AOMI-sleep when compared to AOMI-control (MD 0.37 m/s, CI_95_ 0.16, 0.57, *p* < 0.001 for 10MWT at maximum speed, MD − 1.8 s, CI_95_: − 3.4, − 0.3, *p* = 0.015 for FSST, MD 0.09, CI_95_: 0.01, 0.19, *p* = 0.036 for R-LQYBT in ANT direction) and Control (MD 0.50 m/s, CI_95_ 0.29, 0.71, *p* < 0.001 for 10MWT at maximum speed, MD − 2.4 s, CI_95_ − 3.9, − 0.9, *p* < 0.001 for FSST, MD 0.11, CI_95_ 0.02, 0.20, p = 0.013 for R-LQYBT in ANT direction) at T1. Moreover, AOMI-sleep revealed better 10MWT at self-selected speed (MD 0.18 m/s, CI_95_ 0.03, 0.34, *p* = 0.016) and TUG score (MD − 2.4 s, CI_95_ − 3.8, − 1.0, *p* < 0.001) than Control group, and higher L-LQYBT score in ANT direction (MD 0.10, CI_95_ 0.01, 0.20, *p* = 0.038) than AOMI-control at T1 (Figs. 1 and 2). Within-group post-hoc analysis is showed in Tables 2 and 3.

Furthermore, between-group post-hoc analysis showed better 10MWT at self-selected and maximum speed, TUG and FSST in favor of AOMI-sleep when compared to AOMI-control (MD 0.14 m/s, CI_95_ 0.01, 0.28, *p* = 0.048 for 10MWT at self-selected speed, MD 0.40 m/s, CI_95_ 0.18, 0.63, *p* < 0.001 for 10MWT at maximum speed, MD − 1.5 s, CI_95_ − 2.6, − 0.4, *p* < 0.001 for TUG, MD − 2.1 s, CI_95_: − 3.7, − 0.4, *p* < 0.001 for FSST) and Control (MD 0.26 m/s, CI_95_ 0.12, 0.40, *p* < 0.001 for 10MWT at self-selected speed, MD 0.54 m/s, CI_95_ 0.32, 0.76, *p* < 0.001 for 10MWT at maximum speed, MD − 1.5 s, CI_95_: − 2.7, − 0.4, *p* = 0.006 for TUG, MD − 3.0 s, CI_95_: − 4.6, − 1.4, *p* < 0.001 for FSST) groups at T2. In addition, higher L-LQYBT (MD: 0.13, CI_95_ 0.01, 0.25, *p* = 0.044) and R-LQYBT (MD 0.15, CI_95_ 0.03, 0.26, *p* = 0.047) scores in ANT direction were found in favor of AOMI-sleep when compared to AOMI-control at T2 (Figs. 1 and 2).

Finally, no between-group differences were found for Range-ML during standing in open eyes condition. Within-group post-hoc analysis is showed in Tables 2, 3 and 4.

**Table 4:** Between-group differences over time for static balance outcomes (3 × 3 General Linear Model with Bonferroni post-hoc analysis).

	AOMI-sleep			AOMI-control			Control			*p*-value Time Factor (F-value)	*p*-value Group Factor (F-value)	*p*-value Time x Group interaction (F-value)
	T0	T1	T2	T0	T1	T2	T0	T1	T2			
Open eyes standing												
CoP TOT-PL [mm]	222.4 ± 56.7	200.2 ± 44.4	210.0 ± 61.5	191.5 ± 52.8	183.3 ± 60.0	194.6 ± 66.0	211.2 ± 63.5	230.2 ± 67.6	217.3 ± 68.5	0.721 (0.329)	0.250 (1.423)	0.512 (0.826)
CoP Range-AP [mm]	20.2 ± 5.7	18.6 ± 7.8	20.0 ± 8.0	20.8 ± 5.7	19.0 ± 3.6	17.9 ± 6.8	20.4 ± 5.9	20.1 ± 6.5	21.3 ± 7.7	0.521 (0.657)	0.801 (0.222)	0.536 (0.788)
CoP Range-ML [mm]	27.0 ± 6.1	22.6 ± 6.3	22.4 ± 8.1	22.3 ± 5.8	22.4 ± 5.8	23.9 ± 6.8	22.0 ± 6.4	24.3 ± 6.9	25.0 ± 7.0	0.768 (0.265)	0.816 (0.204)	**0.032** **(2.774)**
Closed eyes standing												
CoP TOT-PL [mm]	367.2 ± 102.9	345.0 ± 102.7	305.4 ± 105.6	299.2 ± 138.0	306.8 ± 145.1	296.9 ± 154.7	326.8 ± 123.9	349.4 ± 107.4	333.6 ± 113.3	0.293 (1.247)	0.630 (0.467)	0.182 (1.599)
CoP Range-AP [mm]	28.3 ± 8.8	25.5 ± 6.2	25.4 ± 8.4	28.8 ± 14.6	26.1 ± 7.7	23.9 ± 9.3	25.1 ± 9.7	27.2 ± 9.2	34.7 ± 36.3	0.838 (0.178)	0.676 (0.394)	0.334 (1.162)
CoP Range-ML [mm]	31.5 ± 14.1	33.2 ± 12.7	29.7 ± 11.6	25.9 ± 12.7	26.5 ± 8.6	26.1 ± 9.3	27.8 ± 7.3	29.0 ± 7.7	25.4 ± 9.0	0.333 (1.114)	0.212 (1.612)	0.926 (0.221)

Data are shown as mean and standard deviation

*AOMI* Action observation plus motor imagery, *CoP* Center of pressure, *TOT-PL* Total path length, *AP* anterior–posterior, *ML* medial–lateral.

Significant *p*-values are shown in bold text.

## Discussion

The study aimed at investigating the effects of early sleep after AOMI on gait and balance performance in older adults. The main findings were that early sleep after AOMI training sessions improved gait and balance abilities in older adults, and these benefits were retained at 1-month after the training end.

The current results agree with studies reporting beneficial effects induced by early sleep on the learning process of trained motor skills in older adults^23,24^. The opportunity enhance gait and balance abilities through the delivery of early sleep after AOMI sessions represents the innovative aspect of this study, since previous literature data have reported limited AOMI effects on gait and balance performance in older adults^18,21^. In addition, the achievement of such sleep-dependent benefits after a training based on the solely systematic observation and imagination of motor tasks in the absence of imitation, further increases the relevance and applicability of this AOMI-training modality. Sleep-dependent motor performance ameliorations have been reported to occur through an offline replay of specific sequences of neural firing at cortical level similar to those experienced during the practice of a motor task. In fact, neurophysiological analyses have reported the occurrence of these neural activity patterns during the slow-wave sleep phase, describing these mechanisms as directly related to motor memory and learning consolidation^22^.

When considering the current study, AOMI-sleep group increased maximum walking speed, exceeding the Minimal Detectable Change of 0.13 m/s, when compared to AOMI-control and Control groups^36^. In addition, maximum walking speed further increased at 1-month follow-up, where these benefits were also extended to self-paced walking speed. The current intervention may be considered in geriatric field, since walking speed represents an index of functional capacity in older adults, as demonstrated by its association with impairments in activities of daily living, cognitive status and risk of fall-related injuries^37,38^. Consistently, AOMI-sleep group improved TUG score, which decreased from baseline to training end. Furthermore, TUG score revealed additional improvements at 1-month follow-up, resulting in significant between-group differences when compared AOMI-control and Control groups. Therefore, positive effects of early sleep after AOMI were not limited to maximal tasks, but sit-to-stand and stand-to-sit performance and the ability to perform a turning task also improved, leading to enhanced mobility. The largest benefits in terms of ability to perform the aforementioned tasks occurred at 1 month after the training end, suggesting that the consolidation of acquired motor skills may take advantage of the reactivation of AOMI contents during sleep. In fact, the physiological consolidation of motor memory traces is a complex process including several time-sensitive consolidation and reconsolidation phases^39,40^. During these phases, memory traces are still susceptible to interference, and the reactivation induced by sleep has been reported to induce significant gains in terms of post-sleep performance^41,42^.

Dynamic balance also improved in AOMI-sleep group compared to AOMI-control and Control groups. In particular, participants allocated to AOMI-sleep group improved FSST score over time and compared to the other groups, which revealed no changes over time. When considering the FSST baseline score, the average scores of all study groups were closer to the cut-off of 10.1 s described by Mathurapongsakul and co-workers for the discrimination between fallers and non-fallers in older adults^43^. After the training and at 1-month after the training end, AOMI-sleep group significantly improved FSST score, which resulted closer to the cut-off of 7.4 s adopted for discriminating between older adults and adults in terms of dynamic balance^43^. LQYBT performed on the left and right lower limbs resulted in higher score in participants performing AOMI followed by early sleep, when compared to subjects who performed the same training at least 12 h before sleeping or the control intervention. Interestingly, between-group improvements exclusively occurred in the ANT direction of the LQYBT. This finding may be related to the features of the AOMI stimuli, which included functional tasks requiring age-matched subjects to perform anterior reaching while maintaining the single-leg stance posture (e.g., kicking balls placed on cones located in front of the subject, wearing shoes placed forward the subject and wearing a sock in single-limb stance posture). Consistently with the current study findings, tasks requiring a reaching in PL or PM directions were not included in visual stimuli, suggesting a task-specificity of balance improvements for the AOMI-training motor contents^31^. The adoption of dynamic balance tasks as AOMI stimuli may also explain the lack of improvements found for static balance abilities. In addition, such lack of effects may also depend on the adopted assessment procedure, since poor sensitivity and high inter-subject variability in terms of center of pressure parameters have been reported during standing tasks in older adults^44^. Finally, no between-group differences over time were found for FES-I. However, participants’ fear of falling was moderate to low, and a ceiling effect cannot be excluded^45^. Moreover, no history of falls was reported by participants and gains achieved in terms of balance abilities may have a limited impact on fear of falling during the activities explored by the FES-I items.

When considering gait and balance improvements experienced by AOMI-sleep group, it is reasonable to speculate that motor learning processes might also be influenced by the time of day in which the training occurred. Literature data has reported fluctuations of physical and mental performance through the day, suggesting that neuroplasticity subtended to motor learning processes may also be influenced by changes induced by circadian rhythms (e.g., variations in cortisol levels)^46^. However, studies have proven that motor performance improvements achieved through physical and/or mental interventions do not seem to be influenced by a particular time of day or diurnal changes in cortisol levels, reinforcing the beneficial role of early sleep on motor skill acquisition experienced by our study participants^46–48^.

Some limitations of the current study need to be underlined. First, although treatment adherence was monitored through a daily phone call and a diary sheet, interventions were unsupervised and differences in terms of attentive status might have influenced the training effects. Second, although visual motor imagery has been described as a viable strategy to enhance motor performance, studies have demonstrated that motor imagery delivered in kinesthetic modality recruits similar brain network to motor execution and induces a corticospinal excitability modulation, when compared to visual motor imagery^49–52^. Therefore, it is reasonable to speculate that the adoption of kinesthetic motor imagery might have further increased the benefits of the training in our participants. In addition, motor imagery abilities were exclusively investigated at baseline, hindering the opportunity to assess potential between-group changes over time. In fact, based on studies showing positive effects of motor imagery practice on imagery capacities and considering the findings of Kaneko and co-workers reporting an increase in motor imagery vividness after a single AOMI session of a walking task, an increase in imaginative skills of our study participants may be expected after the training^18,53^. Moreover, although sleep hours per night were monitored and sleep quality was investigated through the PSQI, the collection of physiological parameters through a polysomnographic monitoring might have provided better sleep monitoring. Finally, neurophysiological investigations might have also provided the brain activity correlates of sleep-dependent gait and balance improvements experienced by AOMI-sleep group.

## Conclusions

Early sleep after AOMI training sessions improved gait and balance abilities in older adults. These findings support the opportunity to adopt this easy-applicable training as a home-based intervention to minimize the functional decay in older adults or boost the effects of an unsupervised balance training without exposing participants to risk of falling.

### Supplementary Information

Supplementary Information.

## Data Availability

The dataset of the study is available from the corresponding author on reasonable request.
