# Supplementary material for: Early sleep after action observation plus motor imagery improves gait and balance abilities in older adults
Source: Sci Rep. 2024 Feb 7;14:3179. doi: 10.1038/s41598-024-53664-2 (PMC10850554; doi:10.1038/s41598-024-53664-2)
Supplement: Supplementary file 1 — Supplementary Information. [file 41598_2024_53664_MOESM1_ESM.docx]

**Early sleep after action observation plus motor imagery improves gait and balance abilities in older adults**

**Authors**: Federico Temporiti, Elena Galbiati, Francesco Bianchi, Anna Maria Bianchi, Manuela Galli, Roberto Gatti

**Supplementary material 1**. Description of video-clips motor contents administered to AOMI-sleep and AOMI-control groups during the 3-week training.

**Week 1**


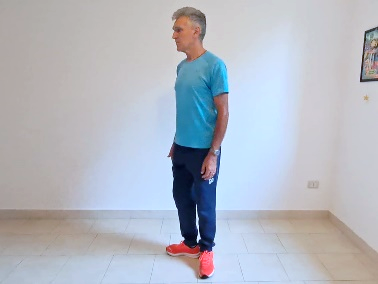

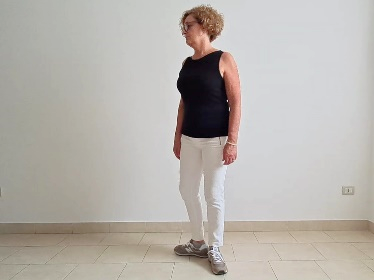


Performing a 360°

rotation (frontal

point of view)


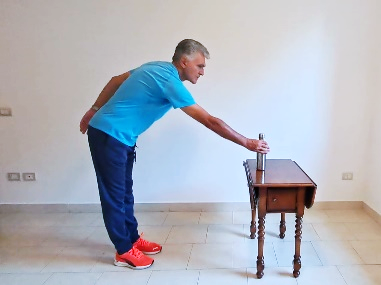

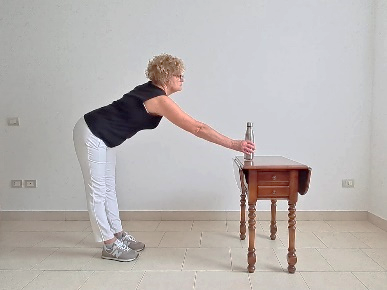


Anterior reaching

of an object (lateral

point of view)


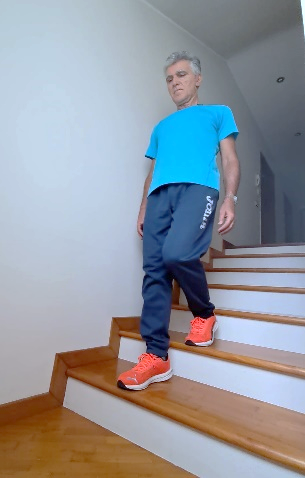

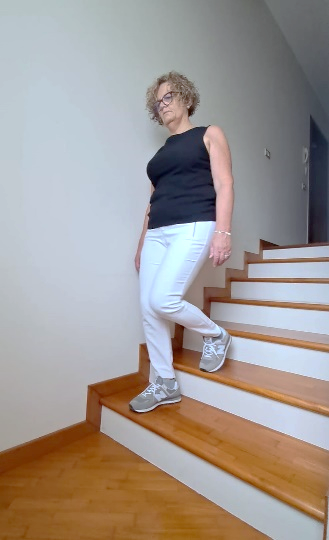


Going down the

Stairs (45° between

frontal and lateral

points of view)

**Week 2**


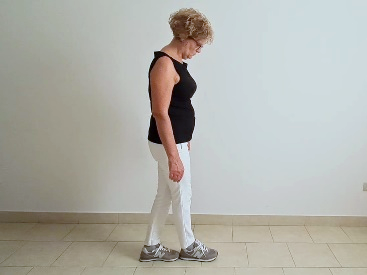

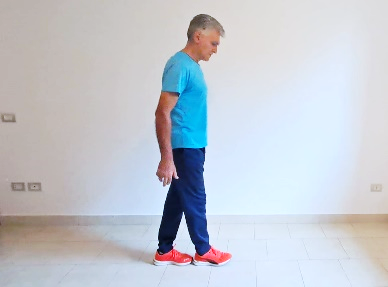

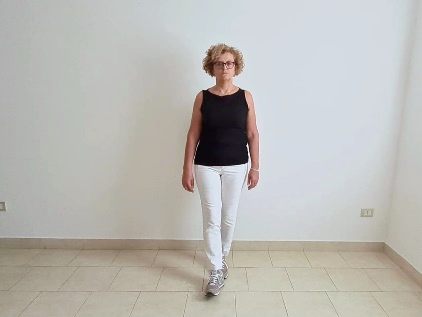


Heel to toe inline

walk (lateral

and frontal points

of view)


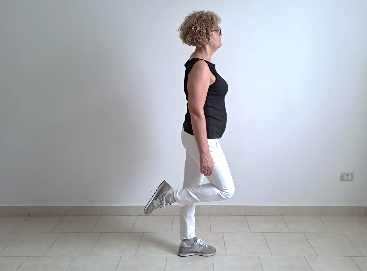

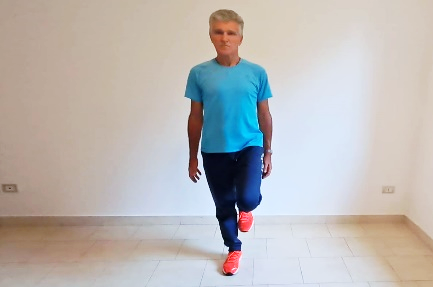

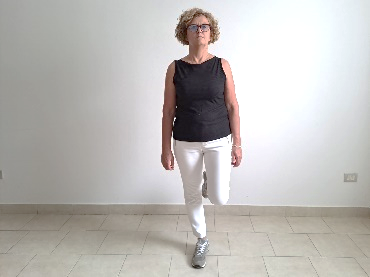


Single-limb stance

on the right and

left lower limbs

(lateral and frontal

points of view)


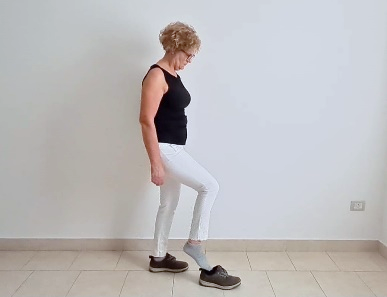

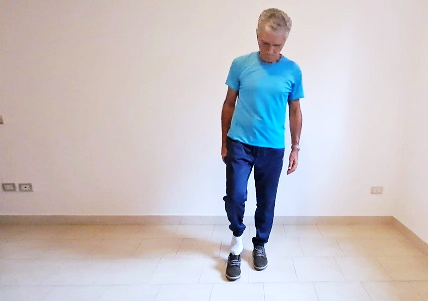

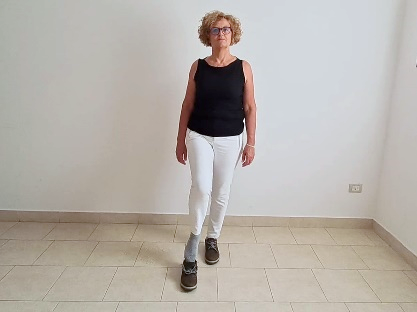


Wearing shoes in

standing posture

(lateral and frontal

points of view)

**Week 3**


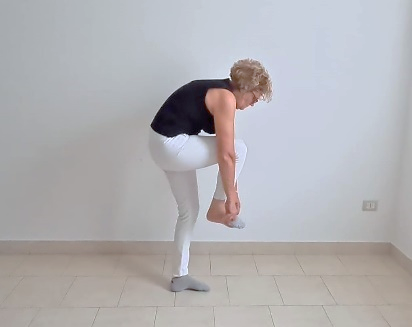

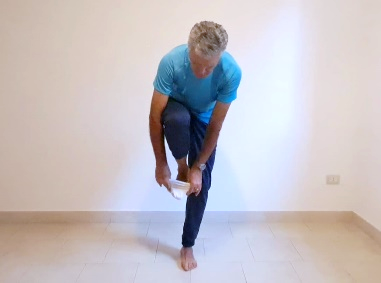

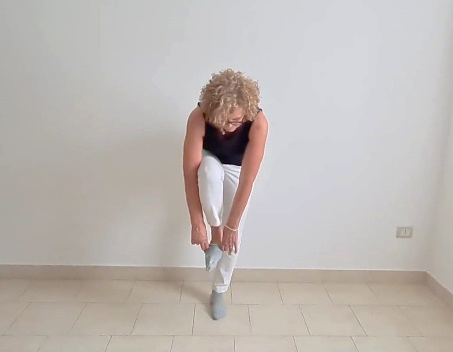


Wearing a sock

in standing posture

(lateral and frontal

points of view)

**
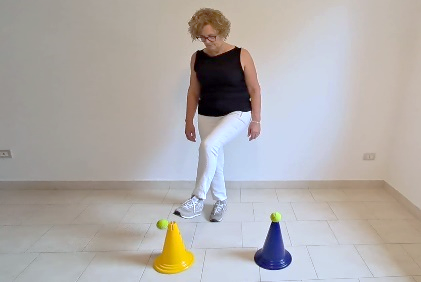
**
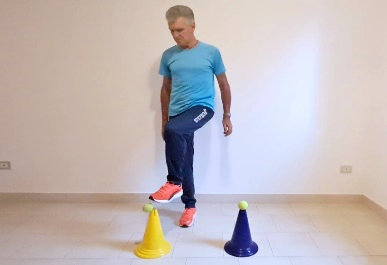
**
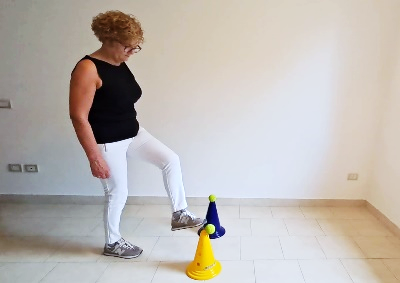
**

Kicking two tennis

balls placed on two

cones (lateral and

frontal points of view)


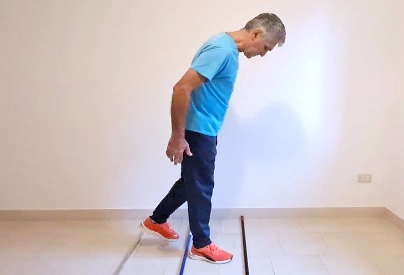

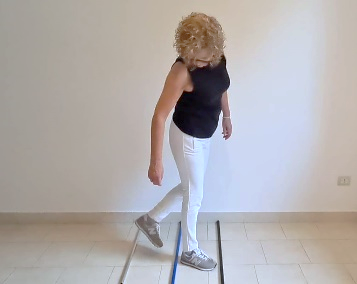


Walking backwards

stepping obstacles

(lateral point of

view)
